# Exploring the microbiome-gut-testis axis in testicular germ cell tumors

**DOI:** 10.3389/fcimb.2024.1529871

**Published:** 2025-01-09

**Authors:** Sona Ciernikova, Aneta Sevcikova, Michal Mego

**Affiliations:** ^1^ Department of Genetics, Cancer Research Institute, Biomedical Research Center of the Slovak Academy of Sciences, Bratislava, Slovakia; ^2^ Department of Oncology, Faculty of Medicine, Comenius University, Bratislava and National Cancer Institute, Bratislava, Slovakia

**Keywords:** the gut microbiome, testicular germ cell tumors, microbiome-gut-testis axis, testicular and seminal microbiome, treatment efficacy

## Abstract

The microbiome-gut-testis axis has emerged as a significant area of interest in understanding testicular cancer, particularly testicular germ cell tumors (TGCTs), which represent the most common malignancy in young men. The interplay between the gut and testicular microbiomes is hypothesized to influence tumorigenesis and reproductive health, underscoring the complex role of microbial ecosystems in disease pathology. The microbiome-gut-testis axis encompasses complex interactions between the gut microbiome, systemic immune modulation, and the local microenvironment of the testis. Dysbiosis in the gut or testicular microbiomes may contribute to altered immune responses, inflammation, and hormonal imbalances, potentially playing a role in the pathogenesis of TGCTs. Concurrently, seminal microbiomes have been linked to variations in sperm quality, fertility potential, and possibly cancer susceptibility, underscoring the need for further evaluation. This review explores the emerging role of the microbiome-gut-testis axis in the context of testicular cancer, highlighting its implications for disease onset, progression, treatment efficacy, and toxicity. Identifying potential microbial biomarkers, followed by microbiota modulation to restore a balanced microbial community, might offer a novel supportive strategy for improving treatment efficacy in refractory TGCT patients while reducing chemotherapy-induced toxicity. We suggest a better understanding of the association between dysregulated microbial environments and TGCTs emphasizes potential pathways by which the gut microbiome might influence testicular cancer.

## 1 Introduction

Testicular cancer is the most common solid malignancy in men aged 20 to 40 (Siegel et al., 2022). Most cases are classified as testicular germ cell tumors (TGCTs), differing from typical solid tumors due to their histological heterogeneity. Advances in treatment, particularly the introduction of cisplatin into therapeutic regimens, have led to significant progress in managing TGCT patients (Einhorn and Donohue, 1977a, b). Despite a high curability, a small subset of patients remains resistant to treatment, prompting extensive research to uncover the underlying mechanisms and identify reliable prognostic biomarkers (Facchini et al., 2019). Current trends in TGCT research comprise efforts to de-escalate therapy in stages with the favorable disease to minimize the acute and long-term treatment-induced toxicity, including the utilization of new approaches in oncosurgery (Franzese et al., 2023). In patients with high-risk disease and relapsed and/or refractory disease, research is primarily focused on overcoming cisplatin resistance and thus increasing the cure rate (Mele et al., 2021).

The gut microbiome exerts a pleiotropic effect on various physiological and pathological processes, including cancer (Nikolaieva et al., 2022). Increasing evidence suggests that microbiome composition modulates the effect of anti-cancer therapy and influences the toxicity of anti-cancer treatment (Ciernikova et al., 2023a). Disruption of intestinal homeostasis is associated with severe diseases and studies have documented the role of specific microorganisms in tumorigenesis (Marshall and Warren, 1984; Ciernikova et al., 2015; Viljoen et al., 2015; D’Antonio et al., 2022; Tan et al., 2022; Ciernikova et al., 2023b). The communication and connection between the gut microbiome and distant organs, such as the brain, muscles, kidneys, and liver, are mediated mainly by microbial signals and metabolites (Chalova et al., 2023). However, studies on testicular tumors are rare. Animal models helped clarify the bidirectional relationship between the gut microbiome and the reproductive system, focusing on the role of androgens. Based on these findings, the microbiome-gut-testis axis has been proposed (Li et al., 2022).

Unfavorable microbiome composition, characterized by the prevalence of pathogens and decreased microbial diversity, negatively affects the efficacy of chemotherapy and immunotherapy (Alexander et al., 2017; Jia et al., 2024). Preclinical and clinical studies documented that tumor-associated microbiome can influence the metabolism and inactivation of anti-cancer drugs (Ciernikova et al., 2022; Fu et al., 2023; Kong et al., 2023; Sevcikova et al., 2023; Abe et al., 2024; Cao et al., 2024). Importantly, gut and tumor microbiome alterations correlated with the response to platinum-based treatment (Iida et al., 2013; Liu et al., 2017; Chambers et al., 2022; Liu et al., 2023). Although cisplatin is part of the first-line chemotherapy regimens given to TGCT patients, minimal data is available regarding gut microbiome analysis in TGCTs.

In this review, we provide the latest knowledge on the role of the gut microbiome in men´s reproductive health, along with study findings on testicular and semen microbiomes. The impact of microbial communities on the efficacy of cisplatin treatment, a first-line therapy for TGCT patients, will also be discussed. Importantly, we will outline possible associations between microbiome composition and TGCT therapeutical outcomes.

## 2 Testicular germ cell tumors

In 2020, a total of 74,500 cases of testicular tumors were diagnosed worldwide, predominantly affecting men of European descent (Sung et al., 2021). Despite the growing incidence of the disease, the mortality rate from testicular cancers has stabilized or shown a declining trend (Huang et al., 2022). In most high- or middle-income countries, cisplatin treatment is widely available, contrasting with limited-resource countries (Cherny et al., 2017). Increased awareness has contributed to earlier diagnosis, improving patient outcomes (McGuinness et al., 2017).

TGCTs account for 95% of all testicular cancers, with a 10-year survival rate surpassing 95% (Hanna and Einhorn, 2014; Fung et al., 2018, 2019). TGCTs can be generally categorized into tumors arising from precursor germ cell neoplasia *in situ* (GCNIS) and GCNIS-unrelated tumors (Rajpert-De Meyts et al., 2016). According to the classification, GCNIS-related tumors comprise two major histologic types: seminomas and non-seminomas. Non-seminomas can be further classified into various subtypes, including embryonal carcinoma, teratoma, or extraembryonic elements such as choriocarcinoma and yolk sac tumors (Oosterhuis and Looijenga, 2019; Paffenholz et al., 2020).

Although the etiology of TGCT remains largely unclear, various genetic and environmental events that occur during fetal testicular development and to some extent, after birth contribute to its onset (Looijenga et al., 2010). Known and potential risk factors include a personal history of cancer in the contralateral testis, a family history of the disease, ethnic differences, body mass index, a diet with high consumption of dairy products, red meat, and baked products, cryptorchidism, age, and precocious puberty, hormonal levels, sex hormone activity, GCNIS, genetic and epigenetic changes, infertility, infections, cigarette and tobacco smoking, and occupational and environmental exposures (Stevenson and Lowrance, 2015; Pallotti et al., 2020; Andelkovic et al., 2023; Crocetto et al., 2023; Yazici et al., 2023; Di Maggio et al., 2024).

## 3 Microbiome-gut-testis axis

The human gut microbiome represents an ecological community of bacteria, viruses, archaea, yeast, fungi, and parasites that colonize the gut (Weiner et al., 2023). The six predominant bacterial phyla that form the microbiome are Bacteroidetes, Firmicutes, Actinobacteria, Proteobacteria, Fusobacteria, and Verrucomicrobia. Among these, Bacteroidetes and Firmicutes collectively comprise about 90% of the gut microbiota (Van Hul et al., 2024), and the balance in Firmicutes/Bacteroidetes ratio is critical for maintaining health (Magne et al., 2020).

Microbiome composition contributes to the regulation of male fertility and reproductive health by influencing testicular function and sperm production (Ma et al., 2024). The microbiome-gut-testis axis is a complex, bidirectional communication system where changes in the gut microbiome can promote systemic alterations and inflammatory responses that negatively affect the testicular environment and sex hormone production (Magill and Macdonald, 2023). Conversely, androgens, important hormones synthesized in the male testes, can influence gut microbiome composition through complex mechanisms (Harada et al., 2016; Li et al., 2022). A healthy lifestyle, diet, supplements, or phytoconstituents support male reproductive health by promoting gut microbiome balance (Dubey et al., 2024). On the other hand, broad-spectrum antibiotics, toxins, endocrine disruptors, and heavy metals can impair intestinal homeostasis, adversely affecting male reproductive health and hormone levels. Gut dysbiosis, characterized by an imbalance in the intestinal microbiota with a prevalence of unfavorable and harmful microorganisms, increases pro-inflammatory markers. The disrupted intestinal barrier facilitates the translocation of pathogens and pro-inflammatory cytokines into the bloodstream (Leelani et al., 2023).

**Figure 1:**
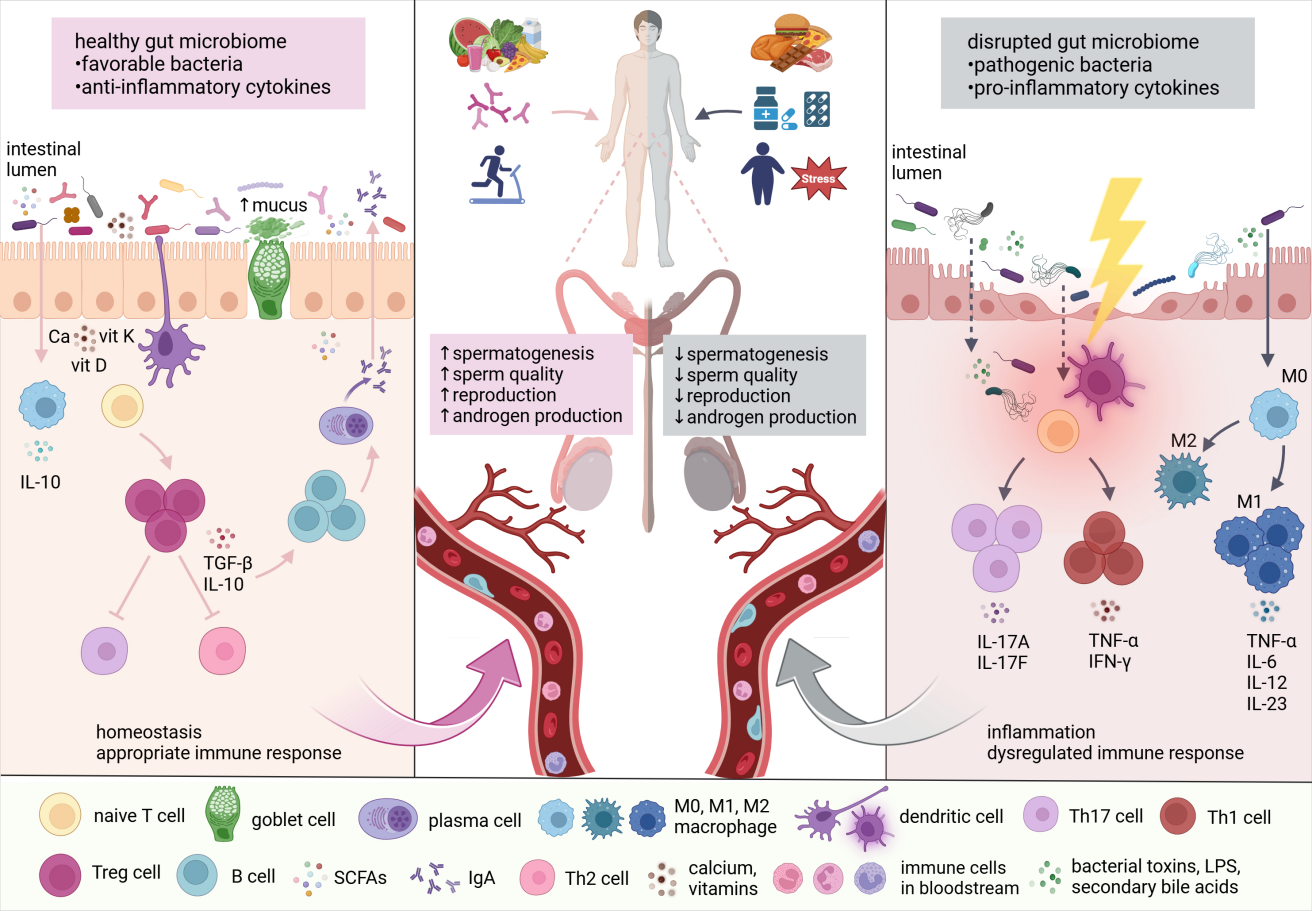
The link between the gut microbiome and testes. Lifestyle factors, including diet, probiotics, physical activity, stress, medications, and toxins significantly influence gut microbiome composition. Subsequently, altered microbial communities affect testicular functions through the production of microbial and microbiota-derived metabolites, vitamins, nutrients, toxins, and reactive oxygen species (ROS). According to numerous findings, the gut microbiome is in direct interaction with the testes, regulating androgen production, spermatogenesis, and overall reproductive capacity (adapted from (Li et al., 2022). Ca, calcium; vit, vitamin; IFN-γ, interferon-gamma; IgA, immunoglobulin A; IL, interleukin; LPS, lipopolysaccharides; M, macrophage; SCFAs, short-chain fatty acids; TGF-β, transforming growth factor-beta; Th cell, helper T cell; TNF-α, tumor necrosis factor-alpha; Treg cell, regulatory T cell.

The reciprocal interactions between the gut microbiome and the testes, proposed as the microbiome-gut-testis axis, highlight the impact of microbiota-derived metabolites on androgen production and metabolism, as well as normal spermatogenesis and reproductive processes (**Figure 1**).

Intestinal microorganisms can modulate androgen metabolism, affecting the androgen levels (Collden et al., 2019). Reciprocally, androgens alter the composition of the gut microbiome by affecting the intestinal barrier and environment (Li et al., 2022). An *in vivo* experiment found no significant differences in the gut microbiome between testosterone-treated mice and control subjects. Despite this, four metabolites, including amino acid derivatives and lipids, were elevated in the testosterone group, while 18 other metabolites showed a decrease (Moadi et al., 2024). Supplementation with *Lactobacillus reuteri* increased testosterone levels in male mice (Lee et al., 2016). Similarly, synbiotics containing *Lactobacillus paracasei* increased testosterone levels in men (Maretti and Cavallini, 2017).

The crosstalk between the gut microbiome and testes has been supported by microbiota modulation with probiotics and fecal microbiota transplantation (FMT), showing fecal transplant from mice on a high-fat diet to normal-diet mice reduced spermatogenesis and decreased sperm motility. *Prevotella copri* was identified as a contributor to impaired sperm motility and increased endotoxin levels in the blood (Ding et al., 2020). Certain probiotics, such as *Lactobacillus rhamnosus* PB01, enhanced sperm dynamics in mice fed a high-fat diet (Dardmeh et al., 2017). Accordingly, *Lactobacillus rhamnosus* NCDC-610 and *Lactobacillus fermentum* NCDC-400 with fructooligosaccharides reduced restraint stress-induced sperm deficits *in vivo*. Moreover, these probiotics demonstrated a protective effect against oxidative stress by decreasing IL-6, IL-10, and TNF-α (Akram et al., 2023). In humans, supplementation with two probiotic strains, *Lactobacillus rhamnosus* CECT8361 and *Bifidobacterium longum* CECT7347, improved sperm motility, and reduced sperm DNA fragmentation in asthenozoospermic males (Valcarce et al., 2017). Similarly, Helli et al. reported enhanced sperm concentration and motility, along with reduced oxidative stress and pro-inflammatory markers, in infertile men supplemented with probiotics (Helli et al., 2022). Yan et al. documented an improved semen quality after fecal transplant of alginate oligosaccharide (AOS)-modified gut microbiota to young mice with type 2 diabetes. Underlying mechanisms included an improved metabolomic profile with elevated levels of butyric acid, docosahexaenoic acid (DHA), eicosapentaenoic acid (EPA), testosterone in the bloodstream and/or testes (Yan et al., 2022).

Disrupted microbial homeostasis affects the production of Vitamins A and K, calcium, and folic acids, potentially leading to impaired testicular function (Cai et al., 2022). A decline in gut absorption of vitamin A was associated with a reduced population of a specific bacterial group, *Ruminococcaceae*_NK4A214. This imbalance led to lower vitamin A levels being delivered to the testes through the bloodstream, which caused damage to sperm production (Zhang et al., 2022). An imbalance in gut bacteria can increase inflammatory signals, triggering immune cells like dendritic cells and macrophages to become more active. These immune cells can enter the testes via the lymphatic system, blood vessels, or other pathways, disrupting the delicate immune environment needed for healthy sperm (Cai et al., 2022). Increased immune activity in the testes and epididymis has been shown to harm sperm development and function (Fijak et al., 2018; Zheng et al., 2021).

## 4 The associations between testicular and seminal microbiomes and reproductive outcomes

Male infertility is one of the known risk factors for testicular cancer (Maiolino et al., 2023). Bryan et al. documented the presence of *Chlamydia trachomatis* in 16.7% of testicular biopsies in infertile men (Bryan et al., 2019). In men with normal sperm count, only small quantities of bacteria were found in testicular specimens using massive ultra-deep pyrosequencing. However, individuals with the absence of sperm in the ejaculate had higher bacterial DNA but decreased microbial diversity, specifically the absence of Bacteroidetes and Proteobacteria (Alfano et al., 2018). Wilharm et al. noted that testicular microbiota influenced the immunoregulatory function of the testes (Wilharm et al., 2021).

A case report involving two male patients with sperm count difficulties described that testicular tissue homogenate from patient with no sperm count contained Firmicutes (53%), Bacteroidetes (12%), Actinobacteria (12%), Proteobacteria (8%), Fusobacteria (8%), SR1 bacteria (7%), Saccharibacteria (3%), and candidate Parcubacteria (1%). The second patient with low sperm count showed high levels of Proteobacteria (64%) in testicular tissue, with lower levels of Firmicutes (13%), Bacteroidetes (15%), and Actinobacteria (7%) (Altun et al., 2021). The testicular microbiome contributes to the bacterial composition of ejaculate. In testicular sperm from the testes of infertile men, ten significantly present bacterial genera were detected, including *Blautia, Cellulosibacter, Clostridium XIVa, Clostridium XIVb, Clostridium XVIII, Collinsella, Prevotella, Prolixibacter, Robinsoniella*, and *Wandonia* (Molina et al., 2021). Another study reported elevated levels of *Aerococcus*, greater seminal α- and β-diversities, and a significant reduction in *Collinsella* in the semen of infertile men (Lundy et al., 2021b).

Brandao et al. suggested the seminal microbiome might affect fertility and seminal quality, and identify an abundance of *Anaerococcus, Bacillus, Burkholderia, Corynebacterium, Finegoldia, Haemophilus, Lactobacillus, Prevotella, Proteus, Pseudomonas, Rhodococcus, Staphylococcus, Streptococcus*, and *Veillonella* in seminal samples (Brandao et al., 2021). Some results indicated no statistically significant differences in seminal microbiome composition between healthy controls and infertile patients, with *Tissierellaceae, Lactobacillaceae*, *Streptococcaceae, Prevotellaceae*, and *Corynebacteriaceae* dominating in both analyzed groups (Amato et al., 2020). On the other hand, numerous microbiome analyses found alterations in the semen microbiome between healthy and infertile men. The underlying mechanisms by which microbes contribute to male infertility, however, remain largely unexplored (Balmelli et al., 1994; Jarvi et al., 1996; Kiessling et al., 2008; Weng et al., 2014; Monteiro et al., 2018; Chen et al., 2023).

A meta-analysis involving 24 studies showed that *Prevotella* negatively impacted sperm quality, while the presence of *Lactobacillus* protected the quality parameters. Increased presence of *Ureaplasma urealyticum* was detected in infertile men (Farahani et al., 2021). Higher levels of metabolite S-adenosylmethionine (SAM) were found in the semen of infertile men. This compound may contribute to infertility because it influences processes like managing oxidative stress, modifying DNA, and supporting cell growth. Changes in SAM levels can negatively affect sperm production and quality. Research also showed that increased levels of *Prevotella* were linked to lower sperm concentration (Lundy et al., 2021a).

## 5 Exploring the role of microbiome in testicular cancer research

The connection between microbiome composition and some genitourinary tumors, including bladder and prostate cancer, has been well established (Kustrimovic et al., 2023; Russo et al., 2024). Signatures of *Helicobacter pylori* were documented in 90% of prostate cancer cases (Banerjee et al., 2019), while higher levels of *Schistosoma, Pseudomonas, Streptococcus, Mycobacterium, Bacteroidetes*, and *Klebsiella* were associated with bladder cancer carcinogenesis (Zhang et al., 2023). However, TGCTs are of embryonic origin, representing a unique entity with distinct biological, clinical, and therapeutic aspects. Thus, findings from other genitourinary cancers cannot be overgeneralized.

Currently, no direct causality has been confirmed between the microbiome and TGCT risk or development. The role of the microbiota in TGCTs has yet to be investigated particularly to its potential involvement through the microbiome-gut-testis axis. Overall, 16S rRNA gene amplicon sequencing and shotgun metagenomic analysis are preferentially used for bacterial taxonomy resolution (Elie et al., 2023). 16S rRNA gene is a microbial biomarker conserved among bacteria, and its sequence contains nine hypervariable regions interspersed with conserved regions (Lan et al., 2016). Shotgun metagenomic analysis offers a strategy to reveal functional metabolic pathways. A small RNA sequencing technique was used to analyze the seminal plasma microbiome from patients with TGCTs or precancerous conditions to explore gene expression profiles, including those of viruses and phages (Morup et al., 2023). *Acaryochloris marina, Burkholderia* spp. *YI23, Halovirus HGTV-1, Thioalkalivibrio* spp. *K90mix*, and *Desulfurivibrio alkaliphilus* had a higher prevalence in patients with TGCTs or precursor lesions compared to controls. Conversely, higher levels of *Streptomyces phage VWB* were found in the control group, suggesting its contribution to healthy testicular development (Morup et al., 2023). In a case study, a patient with testicular seminoma and teratoma experienced notable health benefits after FMT, including normalized stool consistency, decreased levels of anxiety, and an improved ability to tolerate a wide range of foods (Alvaro et al., 2019).

Cancer-testis antigens (CTAs) are normally found only in healthy testes, but their abnormal expression has been observed in various types of cancer (Shim et al., 2023). An *in vitro* experiment demonstrated that the supernatants from *Lactobacillus acidophilus* and *Lactobacillus crispatus* significantly reduced the transcriptional activity of CTAs, specifically ODF4, PIWIL2, RHOXF2, and TSGA10, in cancer cell lines. Recent studies showed that *Lactobacillus* species may downregulate CTA expression through multiple epigenetic mechanisms (Azam et al., 2014). Studies have linked exposure to bisphenol A (BPA) to an increased incidence of testicular cancer (Delbes et al., 2006; Khan et al., 2021). Oral supplementation with *Lactobacillus rhamnosus* and *Lactobacillus plantarum* after BPA exposure effectively removes BPA from the gut, serum, and testes, reduces oxidative stress, and lowers levels of inflammatory cytokines (Wu et al., 2024). In an animal model using Tilapia (*Oreochromis niloticus*), a dietary supplement containing probiotics and vitamin C was shown to mitigate cadmium-induced damage, including bleeding and testicular edema (Hayati et al., 2020).

Yin et al. performed comprehensive microbiome analysis in patients with renal cell and renal pelvis cancer, bladder carcinoma, prostate, and testicular cancer (Yin et al., 2023) using data from the UK biobank (Sudlow et al., 2015) and Finngen consortium (Kurki et al., 2023). According to the findings, an increased risk of testicular cancer was associated with a higher *Peptostreptococcaceae* and the *Romboutsia* genus. On the other hand, the abundance of *Subdoligranulum* correlated with reduced susceptibility to testicular tumorigenesis (Yin et al., 2023). Giampazolias et al. described that an increase in vitamin D modified the gut microbiome to boost cancer immunity (Giampazolias et al., 2024). A clinical study with 120 newly diagnosed or relapsed TGCT patients revealed an association between low plasma vitamin D levels and poor treatment response with higher disease recurrence (Lesko et al., 2023).

**Figure 2:**
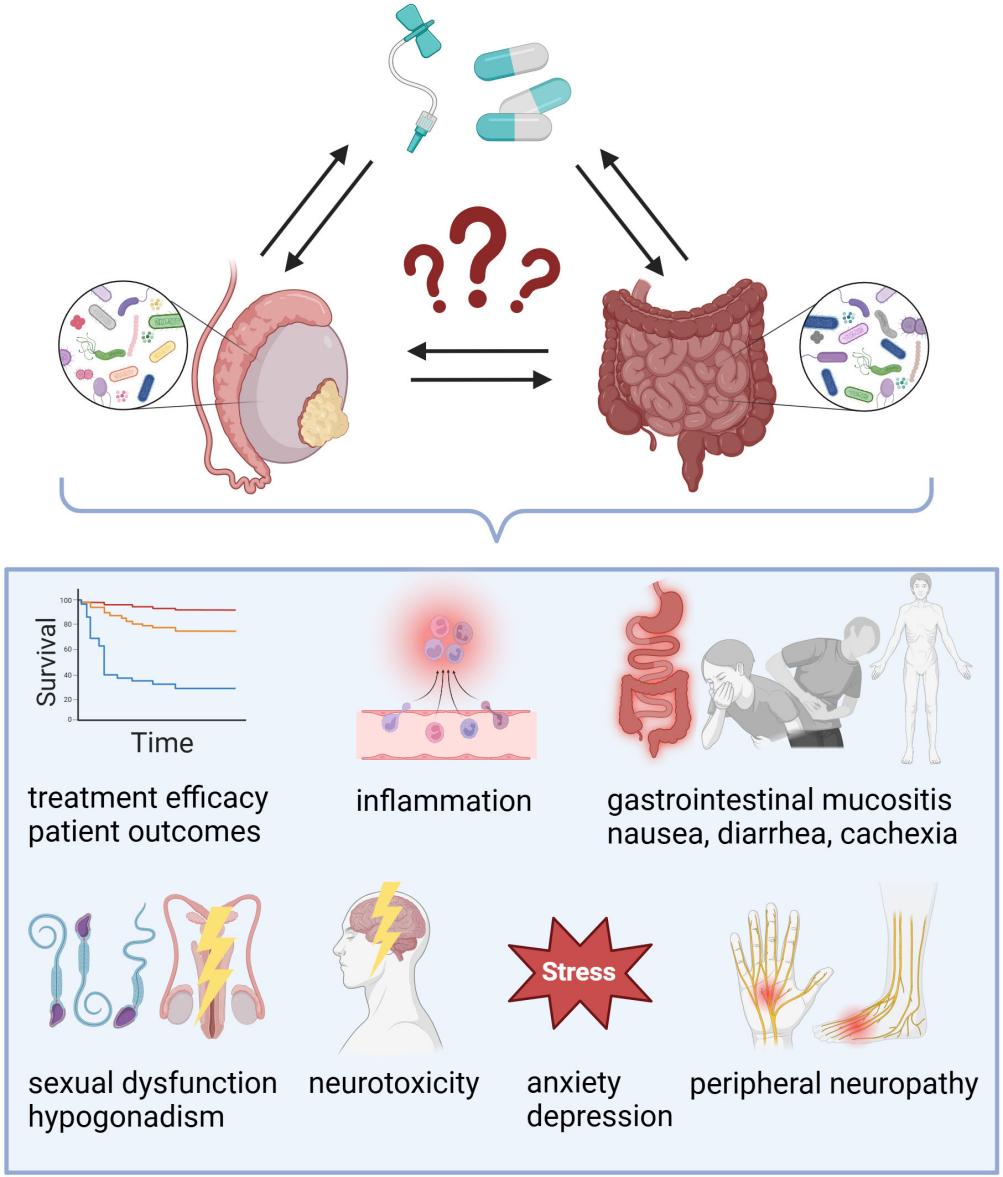
Exploring the impact of gut and testicular microbiomes on treatment outcomes in testicular cancer. While the exact role of microbial communities in testicular germ cell tumors (TGCTs) requires further investigation, existing evidence suggests that the microbiome-gut-testis axis may influence chemotherapy efficacy and contribute to both acute and late treatment-induced toxicity.

Testicular cancer survivors are at risk of experiencing acute and long-term treatment-induced toxicity (**Figure 2**). A study involving 142 TGCT survivors found that patients with higher levels of sCD14, a co-receptor for bacterial lipopolysaccharide (LPS) linked to gut microbial translocation, exhibited reduced cognitive functions compared to those with lower sCD14 levels (Chovanec et al., 2023). Recently, the national, multicenter phase-III registered clinical trial aims to analyze physical activity and cancer-related fatigue in enrolled metastatic TGCT patients treated with cisplatin-based chemotherapy combined with etoposide+/-bleomycin. Moreover, the authors will assess how the gut microbiome affects the connection between physical activities and sequelae (Noh et al., 2024). The long-term goal of the clinical trial NCT05819827 was to investigate associations between chemotherapy-induced nausea and changes in the gut microbiome as well as metabolic pathways in patients with testicular cancer, as well as other genitourinary malignancies such as bladder and prostate cancer. However, this clinical trial has been recently suspended due to lack of funding.

## 6 Discussion and future directions

Emerging evidence highlights the role of the gut microbiome in male infertility, and several associations outline the possible link between the microbiome and TGCTs. However, studies directly analyzing gut or testicular microbiome composition are rare. Identifying microbial biomarkers might help to develop non-invasive screening methods for the analysis of biological material from blood, urine, seminal fluid, and/or stool, that could potentially serve as both diagnostic and prognostic tools for patients with testicular tumors. Moreover, a comprehensive comparison of microbiomes from malignant and benign testicular tissues would bring crucial insights into the dynamics of microbiomes in this area.

Current research struggles to isolate the causal influence of the gut microbiome from confounding factors such as genetics, treatment, and environmental variables. Additionally, the high variability in human microbiome data due to individual differences, together with limitations in sequencing methods, complicates the ability to draw clear conclusions about the microbiome’s functional roles. Most microbiome studies rely on 16S rRNA sequencing, which provides valuable insights into community composition by identifying bacterial taxa but lacks a functional dimension. In contrast, shotgun metagenomic analysis captures genetic information of the entire microbial community, offering critical data on metabolic pathways, virulence factors, and resistance genes. Despite its tremendous potential, standard research and clinical use face significant challenges, including high cost, the need for robust bioinformatics capabilities, and the complexity of interpreting functional profiles for poorly characterized microbial genes. Regardless, a broader application of metagenomic analysis and its combination with other omics approaches could significantly enhance our understanding of dynamic host-microbiome interactions. This comprehensive approach is particularly relevant for personalized medicine, as identifying correlations between microbial functions and specific patient outcomes could facilitate the implementation of more precise and tailored microbiota-based interventions.

Overcoming cisplatin resistance remains a critical hurdle in clinical settings of TGCTs. Research focusing on the personalized analysis of the gut and tumor microbiome composition in TGCT patients could help uncover new microbial biomarkers correlated with disease prognosis and/or toxicity of anti-cancer therapy. A comprehensive investigation of interactions between the microbial community and biological processes associated with tumorigenesis could provide important insights that could help to better predict disease progression, response to therapy, or identify risk factors leading to adverse effects of treatment. Thus, a personalized approach could open up new possibilities not only in predicting treatment outcomes but also in designing targeted interventions aimed at modulating the microbial environment for the benefit of the patient. This is particularly important for cisplatin-treated tumors, given the documented effects of microorganisms on the efficacy of platinum-based chemotherapy.

Several strategies can effectively modulate the gut microbiome, including probiotic supplementation, FMT, dietary changes, and regular physical activity. These approaches can restore microbial diversity and promote intestinal homeostasis. Clinical research involving large patient cohorts could provide relevant insights to enhance the clinical benefits of anti-cancer therapy in TGCT refractory patients. Further investigation is needed to fully understand the interplay between the gut microbiome, the testes, and the testicular and seminal plasma microbiomes, emphasizing the potential benefit for refractory TGCT patients.

## References

[B1] Abe S. Masuda A. Matsumoto T. Inoue J. Toyama H. Sakai A.. (2024). Impact of intratumoral microbiome on tumor immunity and prognosis in human pancreatic ductal adenocarcinoma. J. Gastroenterol. 59, 250–262. doi: 10.1007/s00535-023-02069-5 38242997 PMC10904450

[B2] Akram M. Ali S. A. Kaul G. (2023). Probiotic and prebiotic supplementation ameliorates chronic restraint stress-induced male reproductive dysfunction. Food Funct. 14, 8558–8574. doi: 10.1039/d3fo03153e 37661714

[B3] Alexander J. L. Wilson I. D. Teare J. Marchesi J. R. Nicholson J. K. Kinross J. M. (2017). Gut microbiota modulation of chemotherapy efficacy and toxicity. Nat. Rev. Gastroenterol. Hepatol. 14, 356–365. doi: 10.1038/nrgastro.2017.20 28270698

[B4] Alfano M. Ferrarese R. Locatelli I. Ventimiglia E. Ippolito S. Gallina P.. (2018). Testicular microbiome in azoospermic men-first evidence of the impact of an altered microenvironment. Hum. Reprod. 33, 1212–1217. doi: 10.1093/humrep/dey116 29850857 PMC6012977

[B5] Altun A. Kuran S. B. Keskin İ. Kadioglu A. (2021). The association between the testis microbiota and male infertility. Experimed. 11, 140–142. doi: 10.26650/experimed.2021.955321

[B6] Alvaro Z. T. Hector B.-R. Pedro A. R. L. Silverio A. L. (2019). Testicular cancer and microbiota. GastroMed. Res. 4, 579. doi: 10.31031/GMR.2019.04.000579

[B7] Amato V. Papaleo E. Pasciuta R. Vigano P. Ferrarese R. Clementi N.. (2020). Differential composition of vaginal microbiome, but not of seminal microbiome, is associated with successful intrauterine insemination in couples with idiopathic infertility: A prospective observational study. Open Forum Infect. Dis. 7, ofz525. doi: 10.1093/ofid/ofz525 31915713 PMC6942492

[B8] Andelkovic M. Djordjevic A. B. Vukelic D. Dukic-Cosic D. Acimovic M. Bojanic N.. (2023). Cadmium and lead implication in testis cancer; is there a connection? Chemosphere 330, 138698. doi: 10.1016/j.chemosphere.2023.138698 37062390

[B9] Azam R. Ghafouri-Fard S. Tabrizi M. Modarressi M. H. Ebrahimzadeh-Vesal R. Daneshvar M.. (2014). Lactobacillus acidophilus and Lactobacillus crispatus culture supernatants downregulate expression of cancer-testis genes in the MDA-MB-231 cell line. Asian Pac. J. Cancer Prev. 15, 4255–4259. doi: 10.7314/apjcp.2014.15.10.4255 24935380

[B10] Balmelli T. Stamm J. Dolina-Giudici M. Peduzzi R. Piffaretti-Yanez A. Balerna M. (1994). Bacteroides ureolyticus in men consulting for infertility. Andrologia 26, 35–38. doi: 10.1111/j.1439-0272.1994.tb00751.x 8185059

[B11] Banerjee S. Alwine J. C. Wei Z. Tian T. Shih N. Sperling C.. (2019). Microbiome signatures in prostate cancer. Carcinogenesis 40, 749–764. doi: 10.1093/carcin/bgz008 30794288

[B12] Brandao P. Goncalves-Henriques M. Ceschin N. (2021). Seminal and testicular microbiome and male fertility: A systematic review. Porto Biomed. J. 6, e151. doi: 10.1097/j.pbj.0000000000000151 34881355 PMC8647872

[B13] Bryan E. R. Mclachlan R. I. Rombauts L. Katz D. J. Yazdani A. Bogoevski K.. (2019). Detection of chlamydia infection within human testicular biopsies. Hum. Reprod. 34, 1891–1898. doi: 10.1093/humrep/dez169 31586185 PMC6810529

[B14] Cai H. Cao X. Qin D. Liu Y. Liu Y. Hua J.. (2022). Gut microbiota supports male reproduction via nutrition, immunity, and signaling. Front. Microbiol. 13. doi: 10.3389/fmicb.2022.977574 PMC9434149 36060736

[B15] Cao Y. Xia H. Tan X. Shi C. Ma Y. Meng D.. (2024). Intratumoural microbiota: a new frontier in cancer development and therapy. Signal. Transduction Targeting Ther. 9, 15. doi: 10.1038/s41392-023-01693-0 PMC10776793 38195689

[B16] Chalova P. Tazky A. Skultety L. Minichova L. Chovanec M. Ciernikova S.. (2023). Determination of short-chain fatty acids as putative biomarkers of cancer diseases by modern analytical strategies and tools: a review. Front. Oncol. 13. doi: 10.3389/fonc.2023.1110235 PMC10334191 37441422

[B17] Chambers L. M. Esakov Rhoades E. L. Bharti R. Braley C. Tewari S. Trestan L.. (2022). Disruption of the gut microbiota confers cisplatin resistance in epithelial ovarian cancer. Cancer Res. 82, 4654–4669. doi: 10.1158/0008-5472.CAN-22-0455 36206317 PMC9772178

[B18] Chen P. Li Y. Zhu X. Ma M. Chen H. He J.. (2023). Interaction between host and microbes in the semen of patients with idiopathic nonobstructive azoospermia. Microbiol. Spectr. 11, e0436522. doi: 10.1128/spectrum.04365-22 36633411 PMC9927269

[B19] Cherny N. I. Sullivan R. Torode J. Saar M. Eniu A. (2017). ESMO International Consortium Study on the availability, out-of-pocket costs and accessibility of antineoplastic medicines in countries outside of Europe. Ann. Oncol. 28, 2633–2647. doi: 10.1093/annonc/mdx521 28950323 PMC5834140

[B20] Chovanec M. Kalavska K. Obertova J. Palacka P. Rejlekova K. Sycova-Mila Z.. (2023). Cognitive impairment and biomarkers of gut microbial translocation in testicular germ cell tumor survivors. Front. Oncol. 13. doi: 10.3389/fonc.2023.1146032 PMC10070731 37025582

[B21] Ciernikova S. Mego M. Hainova K. Adamcikova Z. Stevurkova V. Zajac V. (2015). Modification of microflora imbalance: future directions for prevention and treatment of colorectal cancer? Neoplasma 62, 345–352. doi: 10.4149/neo_2015_042 25866215

[B22] Ciernikova S. Sevcikova A. Drgona L. Mego M. (2023a). Modulating the gut microbiota by probiotics, prebiotics, postbiotics, and fecal microbiota transplantation: An emerging trend in cancer patient care. Biochim. Biophys. Acta Rev. Cancer 1878, 188990. doi: 10.1016/j.bbcan.2023.188990 37742728

[B23] Ciernikova S. Sevcikova A. Mladosievicova B. Mego M. (2023b). Microbiome in cancer development and treatment. Microorganisms 12, 24. doi: 10.3390/microorganisms12010024 38257851 PMC10819529

[B24] Ciernikova S. Sevcikova A. Stevurkova V. Mego M. (2022). Tumor microbiome - an integral part of the tumor microenvironment. Front. Oncol. 12. doi: 10.3389/fonc.2022.1063100 PMC9730887 36505811

[B25] Collden H. Landin A. Wallenius V. Elebring E. Fandriks L. Nilsson M. E.. (2019). The gut microbiota is a major regulator of androgen metabolism in intestinal contents. Am. J. Physiol. Endocrinol. Metab. 317, E1182–E1192. doi: 10.1152/ajpendo.00338.2019 31689143 PMC6962501

[B26] Crocetto F. Risolo R. Colapietro R. Bellavita R. Barone B. Ballini A.. (2023). Heavy metal pollution and male fertility: an overview on adverse biological effects and socio-economic implications. Endocr. Metab. Immune Disord. Drug Targets 23, 129–146. doi: 10.2174/1871530322666220627141651 35761486

[B27] D’Antonio D. L. Marchetti S. Pignatelli P. Piattelli A. Curia M. C. (2022). The oncobiome in gastroenteric and genitourinary cancers. Int. J. Mol. Sci. 23, 9664. doi: 10.3390/ijms23179664 36077063 PMC9456244

[B28] Dardmeh F. Alipour H. Gazerani P. van der Horst G. Brandsborg E. Nielsen H. I. (2017). Lactobacillus rhamnosus PB01 (DSM 14870) supplementation affects markers of sperm kinematic parameters in a diet-induced obesity mice model. PloS One 12, e0185964. doi: 10.1371/journal.pone.0185964 29016685 PMC5634625

[B29] Delbes G. Levacher C. Habert R. (2006). Estrogen effects on fetal and neonatal testicular development. Reproduction 132, 527–538. doi: 10.1530/rep.1.01231 17008464

[B30] Di Maggio F. Damaggio G. Nunziato M. Buonaiuto S. Crocetto F. Calabrese A.. (2024). Predictive medicine in a testis trio-family through a combined multi-omics approach. Clin. Transl. Med. 14, e1643. doi: 10.1002/ctm2.1643 38616705 PMC11016938

[B31] Ding N. Zhang X. Zhang X. D. Jing J. Liu S. S. Mu Y. P.. (2020). Impairment of spermatogenesis and sperm motility by the high-fat diet-induced dysbiosis of gut microbes. Gut 69, 1608–1619. doi: 10.1136/gutjnl-2019-319127 31900292 PMC7456731

[B32] Dubey I. Nandheeswari K. Vigneshwaran G. Rohilla G. Lalruatmawii Naxine P.. (2024). Exploring the hypothetical links between environmental pollutants, diet, and the gut-testis axis: The potential role of microbes in male reproductive health. Reprod. Toxicol. 130, 108732. doi: 10.1016/j.reprotox.2024.108732 39395506

[B33] Einhorn L. H. Donohue J. (1977a). Cis-diamminedichloroplatinum, vinblastine, and bleomycin combination chemotherapy in disseminated testicular cancer. Ann. Intern. Med. 87, 293–298. doi: 10.7326/0003-4819-87-3-293 71004

[B34] Einhorn L. H. Donohue J. P. (1977b). Improved chemotherapy in disseminated testicular cancer. J. Urol. 117, 65–69. doi: 10.1016/s0022-5347(17)58338-x 63574

[B35] Elie C. Perret M. Hage H. Sentausa E. Hesketh A. Louis K.. (2023). Comparison of DNA extraction methods for 16S rRNA gene sequencing in the analysis of the human gut microbiome. Sci. Rep. 13, 10279. doi: 10.1038/s41598-023-33959-6 37355726 PMC10290636

[B36] Facchini G. Rossetti S. Berretta M. Cavaliere C. D’aniello C. Iovane G.. (2019). Prognostic and predictive factors in testicular cancer. Eur. Rev. Med. Pharmacol. Sci. 23, 3885–3891. doi: 10.26355/eurrev_201905_17816 31115016

[B37] Farahani L. Tharakan T. Yap T. Ramsay J. W. Jayasena C. N. Minhas S. (2021). The semen microbiome and its impact on sperm function and male fertility: A systematic review and meta-analysis. Andrology 9, 115–144. doi: 10.1111/andr.12886 32794312

[B38] Fijak M. Pilatz A. Hedger M. P. Nicolas N. Bhushan S. Michel V.. (2018). Infectious, inflammatory and ‘autoimmune’ male factor infertility: how do rodent models inform clinical practice? Hum. Reprod. Update 24, 416–441. doi: 10.1093/humupd/dmy009 29648649 PMC6016649

[B39] Franzese D. Tufano A. Izzo A. Muscariello R. Grimaldi G. Quarto G.. (2023). Unilateral post-chemotherapy robot-assisted retroperitoneal lymph node dissection in Stage II non-seminomatous germ cell tumor: A tertiary care experience. Asian J. Urol. 10, 440–445. doi: 10.1016/j.ajur.2023.05.002 38024429 PMC10659970

[B40] Fu A. Yao B. Dong T. Cai S. (2023). Emerging roles of intratumor microbiota in cancer metastasis. Trends Cell. Biol. 33, 583–593. doi: 10.1016/j.tcb.2022.11.007 36522234

[B41] Fung C. Dinh P. Jr. Ardeshir-Rouhani-Fard S. Schaffer K. Fossa S. D. Travis L. B. (2018). Toxicities associated with cisplatin-based chemotherapy and radiotherapy in long-term testicular cancer survivors. Adv. Urol. 2018, 8671832. doi: 10.1155/2018/8671832 29670654 PMC5835297

[B42] Fung C. Dinh P. C. Fossa S. D. Travis L. B. (2019). Testicular cancer survivorship. J. Natl. Compr. Canc. Netw. 17, 1557–1568. doi: 10.6004/jnccn.2019.7369 31805527

[B43] Giampazolias E. Pereira Da Costa M. Lam K. C. Lim K. H. J. Cardoso A. Piot C.. (2024). Vitamin D regulates microbiome-dependent cancer immunity. Science 384, 428–437. doi: 10.1126/science.adh7954 38662827 PMC7615937

[B44] Hanna N. Einhorn L. H. (2014). Testicular cancer: a reflection on 50 years of discovery. J. Clin. Oncol. 32, 3085–3092. doi: 10.1200/JCO.2014.56.0896 25024068

[B45] Harada N. Hanaoka R. Hanada K. Izawa T. Inui H. Yamaji R. (2016). Hypogonadism alters cecal and fecal microbiota in male mice. Gut Microbes 7, 533–539. doi: 10.1080/19490976.2016.1239680 27656762 PMC5153613

[B46] Hayati A. Taufiq A. P. Seftiarini W. Wanguyun A. P. Amira M. Putra P. A. D.. (2020). “The potential of probiotics of malozndialdehide and gonadosomatic index of Tilapia (Oreochromis Niloticus) after exposure of cadmium,” in In IOP Conference Series: Earth and Environmental Science, Vol. 456. 012070. The 10th International Conference on Green Technology (ICGT) "Empowering the Fourth Industrial Revolution through Green Science and Technology" 2-3 October 2019, Malang, Indonesia. Universitas Airlangga. Published under licence by IOP Publishing Ltd. doi: 10.1088/1755-1315/456/1/012070

[B47] Helli B. Kavianpour M. Ghaedi E. Dadfar M. Haghighian H. K. (2022). Probiotic effects on sperm parameters, oxidative stress index, inflammatory factors and sex hormones in infertile men. Hum. Fertil. (Camb) 25, 499–507. doi: 10.1080/14647273.2020.1824080 32985280

[B48] Huang J. Chan S. C. Tin M. S. Liu X. Lok V. T. Ngai C. H.. (2022). Worldwide distribution, risk factors, and temporal trends of testicular cancer incidence and mortality: A global analysis. Eur. Urol. Oncol. 5, 566–576. doi: 10.1016/j.euo.2022.06.009 35863988

[B49] Iida N. Dzutsev A. Stewart C. A. Smith L. Bouladoux N. Weingarten R. A.. (2013). Commensal bacteria control cancer response to therapy by modulating the tumor microenvironment. Science 342, 967–970. doi: 10.1126/science.1240527 24264989 PMC6709532

[B50] Jarvi K. Lacroix J. M. Jain A. Dumitru I. Heritz D. Mittelman M. W. (1996). Polymerase chain reaction-based detection of bacteria in semen. Fertil. Steril. 66, 463–467. doi: 10.1016/S0015-0282(16)58520-3 8751749

[B51] Jia D. Wang Q. Qi Y. Jiang Y. He J. Lin Y.. (2024). Microbial metabolite enhances immunotherapy efficacy by modulating T cell stemness in pan-cancer. Cell 187, 1651–1665 e1621. doi: 10.1016/j.cell.2024.02.022 38490195

[B52] Khan N. G. Correia J. Adiga D. Rai P. S. Dsouza H. S. Chakrabarty S.. (2021). A comprehensive review on the carcinogenic potential of bisphenol A: clues and evidence. Environ. Sci. pollut. Res. Int. 28, 19643–19663. doi: 10.1007/s11356-021-13071-w 33666848 PMC8099816

[B53] Kiessling A. A. Desmarais B. M. Yin H. Z. Loverde J. Eyre R. C. (2008). Detection and identification of bacterial DNA in semen. Fertil. Steril. 90, 1744–1756. doi: 10.1016/j.fertnstert.2007.08.083 18191853

[B54] Kong C. Liang L. Liu G. Du L. Yang Y. Liu J.. (2023). Integrated metagenomic and metabolomic analysis reveals distinct gut-microbiome-derived phenotypes in early-onset colorectal cancer. Gut 72, 1129–1142. doi: 10.1136/gutjnl-2022-327156 35953094

[B55] Kurki M. I. Karjalainen J. Palta P. Sipila T. P. Kristiansson K. Donner K. M.. (2023). FinnGen provides genetic insights from a well-phenotyped isolated population. Nature 613, 508–518. doi: 10.1038/s41586-022-05473-8 36653562 PMC9849126

[B56] Kustrimovic N. Bombelli R. Baci D. Mortara L. (2023). Microbiome and prostate cancer: A novel target for prevention and treatment. Int. J. Mol. Sci. 24, 1511. doi: 10.3390/ijms24021511 36675055 PMC9860633

[B57] Lan Y. Rosen G. Hershberg R. (2016). Marker genes that are less conserved in their sequences are useful for predicting genome-wide similarity levels between closely related prokaryotic strains. Microbiome 4, 18. doi: 10.1186/s40168-016-0162-5 27138046 PMC4853863

[B58] Lee J. Yang W. Hostetler A. Schultz N. Suckow M. A. Stewart K. L.. (2016). Characterization of the anti-inflammatory Lactobacillus reuteri BM36301 and its probiotic benefits on aged mice. BMC Microbiol. 16, 69. doi: 10.1186/s12866-016-0686-7 27095067 PMC4837529

[B59] Leelani N. Bajic P. Parekh N. Vij S. C. Lundy S. D. (2023). The emerging role of the gut-testis axis in male reproductive health and infertility. F&S Rev. 4, 131–141. doi: 10.1016/j.xfnr.2023.01.001

[B60] Lesko P. Vlkova B. Kalavska K. De Angelis V. Novotna V. Obertova J.. (2023). Prognostic role of plasma vitamin D and its association with disease characteristics in germ cell tumours. Front. Oncol. 13. doi: 10.3389/fonc.2023.1149432 PMC10126247 37114140

[B61] Li X. Cheng W. Shang H. Wei H. Deng C. (2022). The interplay between androgen and gut microbiota: is there a microbiota-gut-testis axis. Reprod. Sci. 29, 1674–1684. doi: 10.1007/s43032-021-00624-0 34037957

[B62] Liu D. Hu Y. Guo Y. Zhu Z. Lu B. Wang X.. (2017). Mycoplasma-associated multidrug resistance of hepatocarcinoma cells requires the interaction of P37 and Annexin A2. PloS One 12, e0184578. doi: 10.1371/journal.pone.0184578 28976984 PMC5627893

[B63] Liu Y. Zhou F. Yang H. Zhang Z. Zhang J. He K.. (2023). Porphyromonas gingivalis promotes Malignancy and chemo-resistance via GSK3beta-mediated mitochondrial oxidative phosphorylation in human esophageal squamous cell carcinoma. Transl. Oncol. 32, 101656. doi: 10.1016/j.tranon.2023.101656 36989676 PMC10074990

[B64] Looijenga L. H. Hersmus R. De Leeuw B. H. Stoop H. Cools M. Oosterhuis J. W.. (2010). Gonadal tumours and DSD. Best Pract. Res. Clin. Endocrinol. Metab. 24, 291–310. doi: 10.1016/j.beem.2009.10.002 20541153

[B65] Lundy S. D. Sangwan N. Parekh N. V. Selvam M. K. P. Gupta S. Mccaffrey P.. (2021a). Functional and taxonomic dysbiosis of the gut, urine, and semen microbiomes in male infertility. Eur. Urol. 79, 826–836. doi: 10.1016/j.eururo.2021.01.014 33573862

[B66] Lundy S. D. Vij S. C. Eng C. (2021b). Reply to Eugenio Ventimiglia, Edoardo Pozzi, Massimo Alfano, Francesco Montorsi, and Andrea Salonia’s Letter to the Editor re: Scott D. Lundy, Naseer Sangwan, Neel V. Parekh, et al. Functional and Taxonomic Dysbiosis of the Gut, Urine, and Semen Microbiomes in Male Infertility. Eur. Urol 79, 826–836. doi: 10.1016/j.eururo.2021.04.044 33573862

[B67] Ma Z. Zuo T. Frey N. Rangrez A. Y. (2024). A systematic framework for understanding the microbiome in human health and disease: from basic principles to clinical translation. Signal Transduction Targeting Ther. 9, 237. doi: 10.1038/s41392-024-01946-6 PMC11418828 39307902

[B68] Magill R. G. Macdonald S. M. (2023). Male infertility and the human microbiome. Front. Reprod. Health 5. doi: 10.3389/frph.2023.1166201 PMC10289028 37361341

[B69] Magne F. Gotteland M. Gauthier L. Zazueta A. Pesoa S. Navarrete P.. (2020). The firmicutes/bacteroidetes ratio: A relevant marker of gut dysbiosis in obese patients? Nutrients 12, 1474. doi: 10.3390/nu12051474 32438689 PMC7285218

[B70] Maiolino G. Fernandez-Pascual E. Ochoa Arvizo M. A. Vishwakarma R. Martinez-Salamanca J. I. (2023). Male infertility and the risk of developing testicular cancer: A critical contemporary literature review. Medicina. (Kaunas) 59, 1305. doi: 10.3390/medicina59071305 PMC10383207 37512119

[B71] Maretti C. Cavallini G. (2017). The association of a probiotic with a prebiotic (Flortec, Bracco) to improve the quality/quantity of spermatozoa in infertile patients with idiopathic oligoasthenoteratospermia: a pilot study. Andrology 5, 439–444. doi: 10.1111/andr.12336 28245352

[B72] Marshall B. J. Warren J. R. (1984). Unidentified curved bacilli in the stomach of patients with gastritis and peptic ulceration. Lancet 1, 1311–1315. doi: 10.1016/s0140-6736(84)91816-6 6145023

[B73] McGuinness L. A. Obeidat S. Hickerton B. Long R. (2017). Has increasing public health awareness influenced the size of testicular tumours among adult populations over the last 40 years? J. Public Health (Oxf) 39, 90–94. doi: 10.1093/pubmed/fdw014 26944075

[B74] Mele T. Reid A. Huddart R. (2021). Recent advances in testicular germ cell tumours. Fac. Rev. 10, 67. doi: 10.12703/r/10-67 34557871 PMC8441995

[B75] Moadi L. Turjeman S. Asulin N. Koren O. (2024). The effect of testosterone on the gut microbiome in mice. Commun. Biol. 7, 880. doi: 10.1038/s42003-024-06470-5 39030253 PMC11271554

[B76] Molina N. M. Plaza-Diaz J. Vilchez-Vargas R. Sola-Leyva A. Vargas E. Mendoza-Tesarik R.. (2021). Assessing the testicular sperm microbiome: a low-biomass site with abundant contamination. Reprod. Biomed. Online 43, 523–531. doi: 10.1016/j.rbmo.2021.06.021 34344601

[B77] Monteiro C. Marques P. I. Cavadas B. Damiao I. Almeida V. Barros N.. (2018). Characterization of microbiota in male infertility cases uncovers differences in seminal hyperviscosity and oligoasthenoteratozoospermia possibly correlated with increased prevalence of infectious bacteria. Am. J. Reprod. Immunol. 79, e12838. doi: 10.1111/aji.12838 29500854

[B78] Morup N. Main A. M. Jorgensen N. Daugaard G. Juul A. Almstrup K. (2023). The seminal plasma microbiome of men with testicular germ cell tumours described by small RNA sequencing. Andrology 11, 756–769. doi: 10.1111/andr.13305 36168917

[B79] Nikolaieva N. Sevcikova A. Omelka R. Martiniakova M. Mego M. Ciernikova S. (2022). Gut microbiota-microRNA interactions in intestinal homeostasis and cancer development. Microorganisms 11, 107. doi: 10.3390/microorganisms11010107 36677399 PMC9867529

[B80] Noh H. Anota A. Mongondry R. Meyrand R. Dupuis C. Schiffler C.. (2024). Impact of a one-year supervised physical activity program on long-term cancer-related fatigue and mediating effects of the gut microbiota in metastatic testicular cancer patients: protocol of the prospective multicentre, randomized controlled phase-III STARTER trial. BMC Cancer 24, 84. doi: 10.1186/s12885-024-11824-7 38225551 PMC10790440

[B81] Oosterhuis J. W. Looijenga L. H. J. (2019). Human germ cell tumours from a developmental perspective. Nat. Rev. Cancer 19, 522–537. doi: 10.1038/s41568-019-0178-9 31413324

[B82] Paffenholz P. Pfister D. Heidenreich A. (2020). Testis-preserving strategies in testicular germ cell tumors and germ cell neoplasia in *situ* . Transl. Androl. Urol. 9, S24–S30. doi: 10.21037/tau.2019.07.22 32055482 PMC6995842

[B83] Pallotti F. Pelloni M. Gianfrilli D. Lenzi A. Lombardo F. Paoli D. (2020). Mechanisms of testicular disruption from exposure to bisphenol A and phtalates. J. Clin. Med. 9, 471. doi: 10.3390/jcm9020471 32046352 PMC7074154

[B84] Rajpert-De Meyts E. Mcglynn K. A. Okamoto K. Jewett M. A. Bokemeyer C. (2016). Testicular germ cell tumours. Lancet 387, 1762–1774. doi: 10.1016/S0140-6736(15)00991-5 26651223

[B85] Russo A. E. Memon A. Ahmed S. (2024). Bladder cancer and the urinary microbiome-new insights and future directions: A review. Clin. Genitourin. Cancer 22, 434–444. doi: 10.1016/j.clgc.2023.12.015 38220540

[B86] Sevcikova A. Mladosievicova B. Mego M. Ciernikova S. (2023). Exploring the role of the gut and intratumoral microbiomes in tumor progression and metastasis. Int. J. Mol. Sci. 24, 17199. doi: 10.3390/ijms242417199 38139030 PMC10742837

[B87] Shim K. Jo H. Jeoung D. (2023). Cancer/testis antigens as targets for RNA-based anticancer therapy. Int. J. Mol. Sci. 24, 14679. doi: 10.3390/ijms241914679 37834126 PMC10572814

[B88] Siegel R. L. Miller K. D. Fuchs H. E. Jemal A. (2022). Cancer statistic. CA. Cancer J. Clin. 72, 7–33. doi: 10.3322/caac.21708 35020204

[B89] Stevenson S. M. Lowrance W. T. (2015). Epidemiology and diagnosis of testis cancer. Urol. Clin. North Am. 42, 269–275. doi: 10.1016/j.ucl.2015.04.001 26216814

[B90] Sudlow C. Gallacher J. Allen N. Beral V. Burton P. Danesh J.. (2015). UK biobank: an open access resource for identifying the causes of a wide range of complex diseases of middle and old age. PloS Med. 12, e1001779. doi: 10.1371/journal.pmed.1001779 25826379 PMC4380465

[B91] Sung H. Ferlay J. Siegel R. L. Laversanne M. Soerjomataram I. Jemal A.. (2021). Global cancer statistics 2020: GLOBOCAN estimates of incidence and mortality worldwide for 36 cancers in 185 countries. CA. Cancer J. Clin. 71, 209–249. doi: 10.3322/caac.21660 33538338

[B92] Tan Q. Ma X. Yang B. Liu Y. Xie Y. Wang X.. (2022). Periodontitis pathogen Porphyromonas gingivalis promotes pancreatic tumorigenesis via neutrophil elastase from tumor-associated neutrophils. Gut Microbes 14, 2073785. doi: 10.1080/19490976.2022.2073785 35549648 PMC9116393

[B93] Valcarce D. G. Genoves S. Riesco M. F. Martorell P. Herraez M. P. Ramon D.. (2017). Probiotic administration improves sperm quality in asthenozoospermic human donors. Benef. Microbes 8, 193–206. doi: 10.3920/BM2016.0122 28343402

[B94] Van Hul M. Cani P. D. Petitfils C. De Vos W. M. Tilg H. El-Omar E. M. (2024). What defines a healthy gut microbiome? Gut 73, 1893–1908. doi: 10.1136/gutjnl-2024-333378 39322314 PMC11503168

[B95] Viljoen K. S. Dakshinamurthy A. Goldberg P. Blackburn J. M. (2015). Quantitative profiling of colorectal cancer-associated bacteria reveals associations between fusobacterium spp., enterotoxigenic Bacteroides fragilis (ETBF) and clinicopathological features of colorectal cancer. PloS One 10, e0119462. doi: 10.1371/journal.pone.0119462 25751261 PMC4353626

[B96] Weiner A. Turjeman S. Koren O. (2023). Gut microbes and host behavior: The forgotten members of the gut-microbiome. Neuropharmacology 227, 109453. doi: 10.1016/j.neuropharm.2023.109453 36738776

[B97] Weng S. L. Chiu C. M. Lin F. M. Huang W. C. Liang C. Yang T.. (2014). Bacterial communities in semen from men of infertile couples: metagenomic sequencing reveals relationships of seminal microbiota to semen quality. PloS One 9, e110152. doi: 10.1371/journal.pone.0110152 25340531 PMC4207690

[B98] Wilharm A. Brigas H. C. Sandrock I. Ribeiro M. Amado T. Reinhardt A.. (2021). Microbiota-dependent expansion of testicular IL-17-producing Vgamma6(+) gammadelta T cells upon puberty promotes local tissue immune surveillance. Mucosal Immunol. 14, 242–252. doi: 10.1038/s41385-020-0330-6 32733025 PMC7790758

[B99] Wu J. Zhou T. Shen H. Jiang Y. Yang Q. Su S.. (2024). Mixed probiotics modulated gut microbiota to improve spermatogenesis in bisphenol A-exposed male mice. Ecotoxicol. Environ. Saf. 270, 115922. doi: 10.1016/j.ecoenv.2023.115922 38171106

[B100] Yan X. Feng Y. Hao Y. Zhong R. Jiang Y. Tang X.. (2022). Gut-testis axis: microbiota prime metabolome to increase sperm quality in young type 2 diabetes. Microbiol. Spectr. 10, e0142322. doi: 10.1128/spectrum.01423-22 36214691 PMC9603910

[B101] Yazici S. Del Biondo D. Napodano G. Grillo M. Calace F. P. Prezioso D.. (2023). Risk factors for testicular cancer: environment, genes and infections-is it all? Medicina (Kaunas) 59, 724. doi: 10.3390/medicina59040724 37109682 PMC10145700

[B102] Yin Z. Liu B. Feng S. He Y. Tang C. Chen P.. (2023). A large genetic causal analysis of the gut microbiota and urological cancers: A bidirectional mendelian randomization study. Nutrients 15, 4086. doi: 10.3390/nu15184086 37764869 PMC10537765

[B103] Zhang T. Sun P. Geng Q. Fan H. Gong Y. Hu Y.. (2022). Disrupted spermatogenesis in a metabolic syndrome model: the role of vitamin A metabolism in the gut-testis axis. Gut 71, 78–87. doi: 10.1136/gutjnl-2020-323347 33504491 PMC8666830

[B104] Zhang W. Yang F. Mao S. Wang R. Chen H. Ran Y.. (2023). Bladder cancer-associated microbiota: Recent advances and future perspectives. Heliyon 9, e13012. doi: 10.1016/j.heliyon.2023.e13012 36704283 PMC9871226

[B105] Zheng W. Zhang S. Chen X. Jiang S. Li Z. Li M. (2021). Case report: dendritic cells and macrophages capture sperm in chronically inflamed human epididymis. Front. Immunol. 12. doi: 10.3389/fimmu.2021.629680 PMC7942197 33708220

